# Morphological features and differential counts of *Plasmodium knowles*i parasites in naturally acquired human infections

**DOI:** 10.1186/1475-2875-8-73

**Published:** 2009-04-21

**Authors:** Kim-Sung Lee, Janet Cox-Singh, Balbir Singh

**Affiliations:** 1 Malaria Research Centre, Faculty of Medicine and Health Sciences, Universiti Malaysia Sarawak, 93150 Kuching, Sarawak, Malaysia

## Abstract

**Background:**

Human infections with *Plasmodium knowlesi*, a simian malaria parasite, are more common than previously thought. They have been detected by molecular detection methods in various countries in Southeast Asia, where they were initially diagnosed by microscopy mainly as *Plasmodium malariae *and at times, as *Plasmodium falciparum*. There is a paucity of information on the morphology of *P. knowlesi *parasites and proportion of each erythrocytic stage in naturally acquired human infections. Therefore, detailed descriptions of the morphological characteristics and differential counts of the erythrocytic stages of *P. knowlesi *parasites in human infections were made, photographs were taken, and morphological features were compared with those of *P. malariae *and *P. falciparum*.

**Methods:**

Thick and thin blood films were made prior to administration of anti-malarial treatment in patients who were subsequently confirmed as having single species knowlesi infections by PCR assays. Giemsa-stained blood films, prepared from 10 randomly selected patients with a parasitaemia ranging from 610 to 236,000 parasites per μl blood, were examined.

**Results:**

The *P. knowlesi *infection was highly synchronous in only one patient, where 97% of the parasites were at the late trophozoite stage. Early, late and mature trophozoites and schizonts were observed in films from all patients except three; where schizonts and early trophozoites were absent in two and one patient, respectively. Gametocytes were observed in four patients, comprising only between 1.2 to 2.8% of infected erythrocytes. The early trophozoites of *P. knowlesi *morphologically resemble those of *P. falciparum*. The late and mature trophozoites, schizonts and gametocytes appear very similar to those of *P. malariae*. Careful examinations revealed that some minor morphological differences existed between *P. knowlesi *and *P. malariae*. These include trophozoites of knowlesi with double chromatin dots and at times with two or three parasites per erythrocyte and mature schizonts of *P. knowlesi *having 16 merozoites, compared with 12 for *P. malariae*.

**Conclusion:**

*Plasmodium knowlesi *infections in humans are not highly synchronous. The morphological resemblance of early trophozoites of *P. knowlesi *to *P. falciparum *and later erythrocytic stages to *P. malariae *makes it extremely difficult to identify *P. knowlesi *infections by microscopy alone.

## Background

*Plasmodium knowlesi*, a malaria parasite species commonly found in long-tailed and pig-tailed macaques (*Macaca fascicularis *and *Macaca nemestrina*, respectively) is the only malaria parasite of primates with a 24-hour erythrocytic cycle [1,2]. Humans were shown to be susceptible to *P. knowlesi *by blood passage soon after the parasite was first isolated in 1932 [2] and until recently, naturally acquired human infections with *P. knowlesi *were thought to be extremely rare. However, following a number of reports of human knowlesi malaria infections detected by molecular methods in various countries in Southeast Asia [3-9], *P. knowlesi *is now recognized as the fifth species of *Plasmodium *infecting humans [10].

Malaria diagnosis relies heavily on examination of stained blood films by microscopy, leading to parasite detection, enumeration and identification of *Plasmodium *species for rational decisions on appropriate patient treatment and management. The parasite species is identified by morphological characteristics and for the diagnosis of human malaria, detailed morphological descriptions for *Plasmodium falciparum, Plasmodium vivax, Plasmodium malariae *and *Plasmodium ovale *are given in medical text books and malaria diagnostic reference literature [11,12]. Morphological descriptions of *P. knowlesi *in the parasitology literature are largely based on experimental infections of rhesus macaques, since *P. knowlesi *causes a high parasitaemia in these hosts, as opposed to low parasitaemia in its natural macaque hosts [1,13,14]. There are no detailed descriptions of the morphology of *P. knowlesi *in naturally-acquired human infections other than the description by Singh *et al *[3] in which it was reported that the early trophozoites resemble *P. falciparum *while the other stages are similar to those of *P. malariae*. These morphological similarities between *P. knowlesi, P. malariae *and *P. falciparum *have been noted previously by other workers but without accompanying detailed accounts with coloured photographs [1,15,16]. It remains unclear whether *P. knowlesi *parasite stages in human erythrocytes are truly indistinguishable from *P. falciparum *and *P. malariae *infections by microscopy. Therefore, the morphology of the different blood stages of *P. knowlesi *parasites observed in human erythrocytes from knowlesi malaria patients is described, together with quantitative estimates of the different erythrocytic stages, and compared with those of the other malaria parasites causing human disease.

## Methods

### Patient details and collection of blood films

Thick and thin blood films from ten patients admitted to Kapit hospital and subsequently confirmed by nested PCR assays [3] as having single *P. knowlesi *infections were randomly selected from films that had been prepared from 83 knowlesi malaria patients admitted to Kapit Hospital [4]. These patients were aged between 14 to 65 years, and 6 were males (patients numbered KH364, KH370, KH399, KH412, KH421, KH433) and 4 were females (KH328, KH369, KH422, KH431). Blood films were prepared at the time of admission prior to commencement of anti-malarial treatment. Informed verbal consent was obtained from each patient before blood samples were taken.

### Staining of thick and thin blood films

Thin blood films were fixed with absolute methanol (BDH, England) for 10 seconds and were allowed to dry at room temperature before staining. Thick and thin blood films were stained with 3% and 10% Giemsa (BDH, England) respectively, in Gurr^® ^buffered water, pH 7.2 (BDH, England) for 30 minutes, as described previously [11]. All thick and thin blood films were examined under the microscope at a magnification of × 1,000 with immersion oil.

### Parasite counts and determination of differential parasitaemia

The parasitaemia of each patient in the study was calculated from thick blood films by counting the number of parasitized red blood cells per white blood cells in over 100 fields and calculated using the actual white blood cell count of each patient. The percentage of each of the parasite developmental stages was determined based on the number of early trophozoites, late and mature trophozoites, schizonts, and gametocytes in thin blood films. A total of 500 parasitized erythrocytes were counted for each of the 10 patients except for patients KH328 and KH422 where 50 parasitized erythrocytes were counted due to the low parasitaemia. The morphology of each of *P. knowlesi *parasite developmental stages was scored according to the descriptions by Garnham [1] and Coatney *et al *[16] as follows: early trophozoite – parasite appears as ring of cytoplasm with chromatin dot and no malaria pigment; late and mature trophozoites – parasites with denser cytoplasm, undivided nuclear chromatin, with or without malaria pigment; schizont – parasite with multiple masses of nuclear chromatin and clumps of dark brown pigment; gametocytes – parasites appear to fill most of the host erythrocyte with single large chromatin mass and scattered prominent pigment. In addition, other characteristics such as multiple infections of single erythrocytes with early and mature trophozoites, the number of chromatin dots in ring-form stages and descriptions on the abundance and location of malaria pigment were recorded. The sizes of the parasites were measured using a measuring eyepiece graticule at 1,000× magnification. The sizes of infected erythrocytes were measured relative to surrounding uninfected erythrocytes.

## Results

### Differential counts and morphological characteristics of *P. knowlesi *parasites

**Figure 1:**
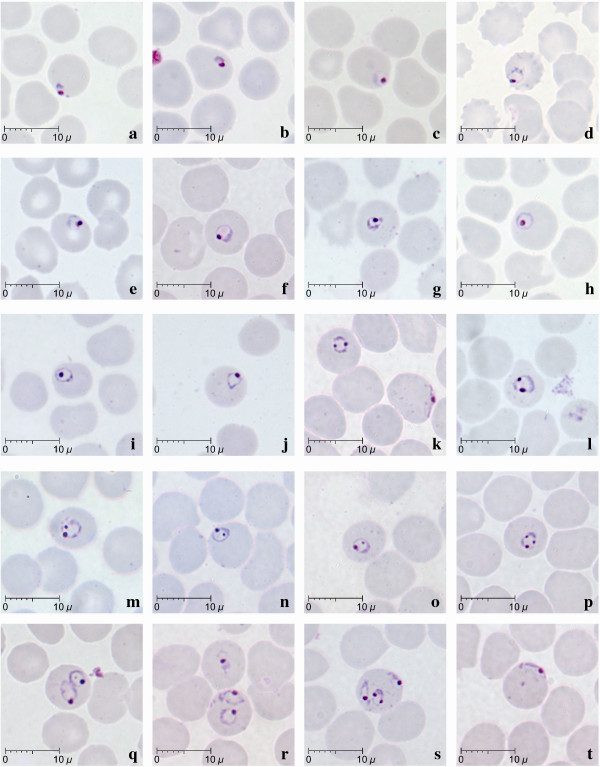
**Early trophozoites of *P. knowlesi *parasites in human infections**. Giemsa-stained thin blood films from patients KH369-a,c,f,k,m,n,o,p,r,s,t; KH370-d,h; KH399-b; KH412-l, KH421-e,j; KH433-g,i,q.

**Figure 2:**
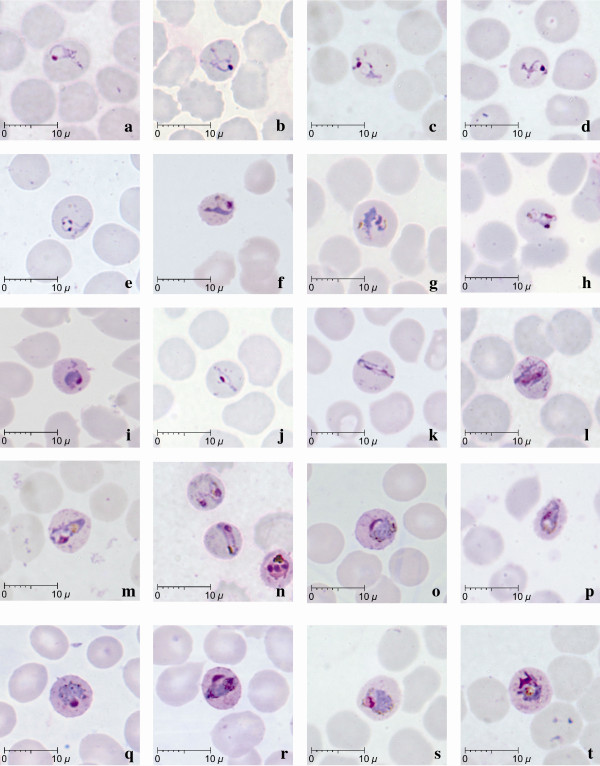
**Late (a-l) and mature (m-t) trophozoites of *P. knowlesi *parasites in human infections**. Giemsa-stained thin blood films from patients KH364-n; KH369-a,g,l,s; KH370-b,j; KH399-h,p; KH412-c,m,t; KH421-f,i; KH422-o,q,r; KH433-d,e; KH431-k.

**Figure 3:**
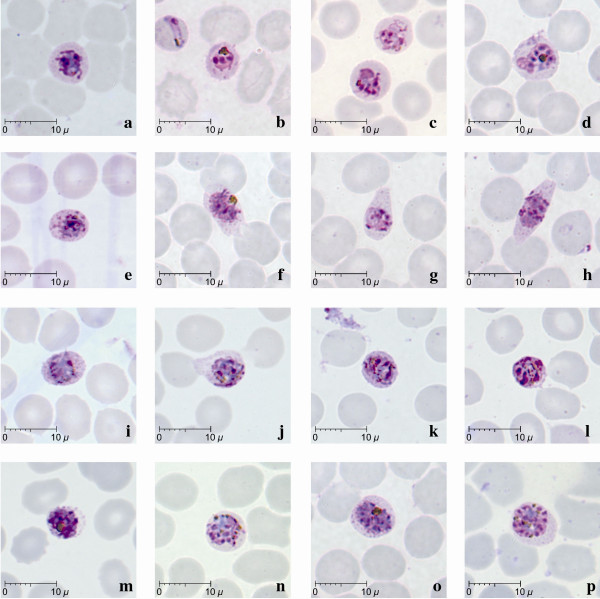
**Schizonts of *P. knowlesi *parasites in human infections**. Giemsa-stained thin blood films from patients KH364-b; KH369-c,d,f,g,h,n,o,p; KH412-a; KH421-j,m; KH422-e,i; KH431-k,l.

**Figure 4:**
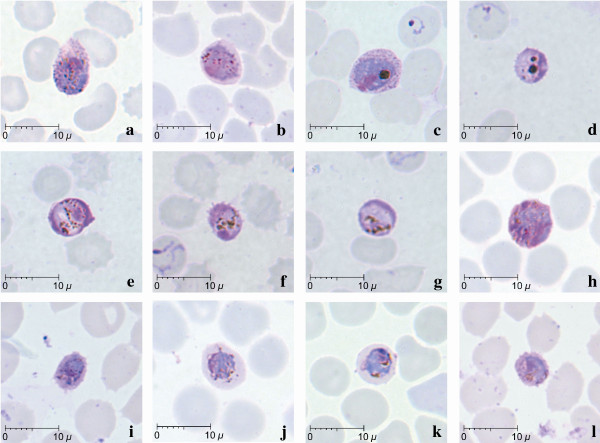
**Gametocytes of *P. knowlesi *parasites in human infections**. Giemsa-stained thin blood films from patients KH370-a,h; KH369-b,c,j,k; KH364-d,e,f,g; KH431-i,l.

**Figure 5:**
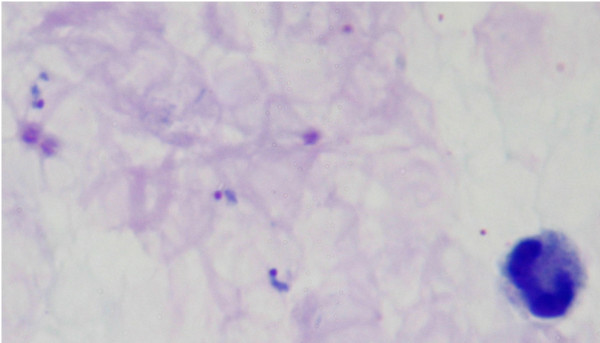
**Giemsa-stained thick blood film from patient KH433 showing late trophozoites of *P. knowlesi***.

Thick and thin blood films were examined from ten patients with *P. knowlesi*, with a parasitaemia between 610 to 236,000 parasites per μl blood (Table 1). All of the developmental stages of the malaria parasite life cycle expected in the peripheral blood were observed in Giemsa-stained thin blood films and are presented (Figures 1, 2, 3, 4 and 5). The infections in all 10 patients were asynchronous, except for one patient (KH433) who had 97% of parasites in the late trophozoite stage without malaria pigment at the time of admission (Table 1; Figure 1g, i, q; Figure 5).

**Table 1:** Differential parasitaemia in 10 patients with single infections of *P. knowlesi*.

		**Percentages (%)**			
		
Patient code	Parasitaemia (parasites per μl blood)	Early trophozoites	Late/Mature trophozoites	Schizonts	Gametocytes
KH433	7,708	2.0	97.0	1.0	0
KH421	10,290	77.0	17.0	6.0	0
KH370	1,908	72.1	26.1	0	1.8
KH328	610	66.7	33.3	0	0
KH369	9,751	34.4	20.9	43.2	1.5
KH399	9,700	31.0	65.0	4.0	0
KH412	2,752	29.0	67.0	4.0	0
KH431	1,155	25.6	61.7	11.5	1.2
KH364	*236,000	4.4	82.1	10.7	2.8
KH422	920	0	76.0	24.0	0

*based on examination of thin blood film

Early trophozoites of *P. knowlesi *were seen in all patients examined except one (KH422). They were characterized by the appearance of a ring-like cytoplasm enclosing a vacuole with a dot of round nuclear chromatin projecting from the cytoplasm (Figure 1c–g). Some early trophozoites with small and non-vacuolated cytoplasm appeared as a mass of bluish stained bodies measuring between 1.5 to 2 μm (Figure 1, a–b). The ring form trophozoite of *P. knowlesi *measured approximately one third to half the diameter (2.5 to 4 μm) of the infected erythrocyte (Figure 1, c–s). The cytoplasm of the ring form trophozoite was slightly thicker especially on the side opposite the nuclear chromatin dot (Figure 1, c–g). A single prominent nuclear chromatin dot (Figure 1, a–j, ) was common (62.5 to 100% of ring forms in all nine patients with early trophozoites; Table 2) though double chromatin dots (Figure 1, k–o) were also frequently observed (7.8 to 37.5% of ring forms in 8 of 9 patients with early trophozoites; Table 2). Double chromatin dots were mostly situated at opposing poles of the ring form (Figure 1, k–l) but occasionally seen to be closely together (Figure 1m). In addition, a single or accessory chromatin dot lying within the vacuole was occasionally seen (Figure 1,h, i, n, o). Early trophozoites with triple chromatin dots were rare and only seen in one of the patients (Table 2; Figure 1, p). Multiply-infected erythrocytes were observed in blood films from three of the 10 patients, of which only one (KH364) had a parasitaemia above 100,000 parasites/μl blood. Single erythrocytes containing more than three parasites were observed in two patients (Table 2; Figure 1, s), and doubly infected erythrocytes (Figure 1, q, r) was the more common form of multiple infections in three patients (Table 2). Appliqué forms, that resembled those of *P. falciparum*, were infrequently seen, being observed in only three patients (Figure 1, k, s, t; Table 2). In all erythrocytes containing early trophozoites of *P. knowlesi*, enlargement of erythrocytes and stippling were not observed.

**Table 2:** Characteristics of *P. knowlesi *parasites in naturally acquired human infections.

	Malaria pigment (%)		*Chromatin dots (%)			**Number of parasites in single erythrocyte (%)			Band form trophozoite	Appliqué form
			
Patient code	Clumped	Scattered	Single	Double	Triple	1	2	3		
KH369	81.5	18.5	71.6	25.8	2.6	92.4	6.8	0.8	Yes	Yes
KH364	75.0	25.0	92.2	7.8	0	74.9	20.6	4.5	Yes	Yes
KH370	n	n	88.9	11.1	0	100.0	-	-	Yes	-
KH412	100.0	0	62.5	37.5	0	100.0	-	-	Yes	Yes
KH433	n	n	85.7	14.3	0	100.0	-	-	-	-
KH431	5.6	94.4	89.3	10.7	0	98.2	1.8	-	Yes	-
KH421	16.7	83.3	81.3	18.7	0	100.0	-	-	-	-
KH422	0	100	0	0	0	100.0	-	-	Yes	-
KH328	0	100	85.7	14.3	0	100.0	-	-	-	-
KH399	33.3	66.7	100.0	0	0	100.0	-	-	-	-

*based on early trophozoites or ring form stages, and these were absent in patient KH422.

**based on early and late trophozoite stages.

n = no pigment because no mature trophozoites were observed.

Late and mature trophozoites were seen in all 10 patients examined and were characterized by denser cytoplasm compared to the ring-like cytoplasm in early trophozoites (Figure 2, a–t). The cytoplasm of the late trophozoites of *P. knowlesi *appeared to be slightly amoeboid and irregular in shape, while vacuoles were maintained (Figure 2, a–h). Late trophozoites measured approximately 3 to 5 μm. The cytoplasm of the parasites that extended across the erythrocyte forming "band-like" trophozoites was seen in six of the 10 patients (Figure 2, g, h, k, l, r; Table 2). Nuclear chromatin dots of late trophozoites seemed to be slightly larger in size compared to those in the early trophozoites (Figure 2). There was very little or no obvious malaria pigment in the majority of the late trophozoites stages.

Mature trophozoites of *P. knowlesi *appeared slightly larger compared to the late trophozoite with more solid and dense cytoplasm measuring approximately 5 to 6 μm and the presence of malaria pigment. The nuclear chromatin mass was more conspicuous but remained undivided (Figure 2, l–t) and vacuoles were small or completely absent (Figure 2, m–t). Malaria pigment was observed either in the form of scattered, fine dark brown grains (18.5 to 100% of infected erythrocytes; Table 2, Figure 2, g, h,l, m, o, p, s), or clumps of dense golden brown granules (5.6 to 100% of infected erythrocytes; Table 2, Fig 2, n, q, r, t) in mature trophozoites. Infected erythrocytes were generally not enlarged and stippling such as more recent entomological Schüffner's dots was not observed. However, irregular and sparse dots that were unevenly distributed were noticed in some infected erythrocytes with mature trophozoites (Figure 2, l, n, r, t).

Young and mature schizonts were seen in eight of the 10 patients examined, though they were found to be less common in three of these patients (<5% of total infected erythrocytes, Table 1). Young schizonts, characterized by the appearance of between 2 to 5 divided nuclear chromatin masses and abundant pigment granules, were common, occurring in eight of the 10 patients (Figure 3, a–i). They occupied at least two thirds (5 to 6 μm) of the infected erythrocyte while mature schizonts appeared to occupy nearly the whole infected erythrocyte. A maximum number of 16 merozoites in mature schizonts was observed in the blood film from patient KH369 (Figure 3, p). The divided chromatin masses and merozoites in young and mature schizonts were either irregularly scattered (Figure 3, l, m, n) or in the form of a grape-like cluster (Figure 3, o, p). Malaria pigment in both young and mature schizonts appeared to be either aggregated into many small granules (Figure 3, i, j, k, l, n, p ) or aggregated into dense clumps of brownish black round mass (Figure 3, b, d, e, f, m, o). The position of pigment aggregates was variable. Infected erythrocytes with mature schizonts were not enlarged compared to uninfected erythrocytes. Distorted and fimbriated infected erythrocytes were observed rarely in the blood film from only one patient, KH369 (Figure 3, f, g, h). Fine irregular stippling was evident in most of the erythrocytes containing schizonts (Figure 3, a, e–j, o, p), where the parasites occupied approximately two thirds of the erythrocyte.

Gametocytes, comprising only between 1.2 to 2.8% of infected erythrocytes, were observed in four of the 10 patients examined (Figure 4; Table 2). Mature gametocytes were characterized by their spherical shape occupying most of the infected erythrocyte, chromatin was compact and the dark brown pigment was irregularly scattered (Figure 4, a–h). Young gametocytes occupied about two thirds of the infected erythrocytes and were difficult to differentiate from mature trophozoites (Figure 4 i–l). The cytoplasm of macrogametocytes appeared bluish with the dense pinkish chromatin situated at the periphery of the parasite (Figure 4 a–d). In contrast, the cytoplasm of microgametocytes stained a pinkish-purple hue with a variably positioned darker large chromatin mass (Figure 4 e–h). Pigment grains in both macro and microgametocytes were seen to be irregularly distributed (Figure 4 a, b, e–l), however, macrogametocytes with clumps of dense dark-brownish malaria pigment were also observed (Figure 4 c–d). The infected erythrocytes were generally not enlarged, though some gametocytes were seen to be slightly smaller than the uninfected erythrocytes (Figure 4 d, f, i, l).

### Morphological comparison between *P. knowlesi *and *P. malariae*

The morphology of *P. knowlesi *parasites and the characteristic of the infected erythrocyte in human infections in the present study were compared with those of *P. malariae *as described in the literature [1,12,16] (Table 3).

**Table 3:** Comparison between *P. knowlesi *and *P. malariae *parasites in human infections

	P. malariae*	P. knowlesi
**HOST ERYTHROCYTE**		
Size	Not enlarged	Not enlarged
Shape	Rounded, not distorted	Rounded, generally not distorted
Stippling	Ziemen's stippling under certain staining conditions	Irregular dots or stippling in some erythrocytes with mature trophozoites, schizonts and gametocytes
		
**PARASITE**		
Early trophozoite (ring form)	Ring form with dense cytoplasm and single chromatin; sometimes with accessory dot	Ring forms are compact with dense cytoplasm; single or double chromatin and rarely triple chromatin; appliqué forms; multiple parasites in single erythrocyte
Late trophozoite	Regular and compact cytoplasm; appearance of pigment	Dense and thick cytoplasm; Cytoplasm slightly amoeboid and irregular; band forms; varying pigmentation
Mature trophozoite	Compact, rounded, heavily pigmented; band forms; not amoeboid	Compact and dense cytoplasm, rounded shape with dark brown pigment; band forms; not amoeboid
Schizont	Occupies whole erythrocyte, contains 6–12 merozoites, usually 8; merozoites clustered around dark-brown malaria pigment forming rosette pattern	Occupies whole erythrocyte; contains up to 16 merozoites; merozoites irregularly scattered or grape-like cluster; malaria pigment scattered or collects into a single mass
Gametocyte	Round, compact, occupies whole erythrocyte, scattered malaria pigment; early forms are very similar to mature trophozoites	Round, compact, fills whole erythrocyte, scattered or clumped malaria pigment; early forms are very similar to mature trophozoites

*Based on descriptions by Garnham [1], Coatney *et al *[16] and Warrell & Gilles [12].

## Discussion

*Plasmodium knowlesi *was first isolated in 1931 in India from a long-tailed macaque imported from Singapore [2]. It was studied in a number of different primate species and was also shown to be infectious to humans via blood passage in 1932 [1,2]. The detailed morphology of *P. knowlesi *was described by Sinton and Mulligan [14], as observed in infections in rhesus monkeys. Here, the morphological descriptions of *P. knowlesi *malaria parasites from naturally acquired human infections, that had all been misdiagnosed by microscopy as *P. malariae*, are described.

Detailed observations of Giemsa-stained thin blood films from 10 patients with varying degree of parasite load, showed that human infections were generally asynchronous at the time of admission with all stages of the erythrocytic cycle of *P. knowlesi *parasites present in seven of the 10 patients. Only one patient (KH433) had a highly synchronous infection with 97% of infected erythrocytes containing late trophozoites.

Early trophozoites of *P. knowlesi *appeared as ring forms within the infected erythrocyte and possessed characteristics that resembled the early trophozoites of *P. falciparum *such as double chromatin dots, multiply-infected erythrocytes and appliqué forms. The resemblance of the early trophozoites of *P. knowlesi *to those of *P. falciparum *has also been noted previously in human *P. knowlesi *infections [3,15] and in experimental infections in rhesus monkeys [2,14]. Human infections with *P. knowlesi *can be easily mistaken for falciparum malaria when the infecting parasites are predominantly at the early trophozoite or ring form developmental stage. If the blood film had been prepared earlier from the patient with the highly synchronous infection, most of the trophozoites would have appeared as ring forms and the infection would have been diagnosed by microscopy as *P. falciparum*.

Late and mature trophozoites, schizonts and gametocytes of *P. knowlesi *in human infections were generally indistinguishable from those of *P. malariae*. There were no specific features of the cytoplasm, nucleus and pigment of the parasites or the infected erythrocytes that could easily distinguish *P. knowlesi *from *P. malariae*, other than the presence of very amoeboid cytoplasm. However, this was observed in only some late trophozoites of *P. knowlesi*. Moreover, 'band form' trophozoites, which are a characteristic feature for *P. malariae *parasites [1,16,17] were observed in more than half of the blood films examined.

In agreement with previous descriptions of the number of merozoites in mature *P. knowlesi *schizonts in infections of rhesus macaques [1,14,16], a maximum of 16 merozoites per mature schizont were observed in one of the human infections, which is higher than the 12 reported for a mature schizont of *P. malariae *[1,16,17]. In addition, no mature schizonts of *P. knowlesi *were observed with symmetrical arranged merozoites surrounding clumps of malaria pigments or "rosette pattern," which was often described as a characteristic of schizonts of *P. malariae *[1,16,17].

The infected erythrocytes for all stages were generally not enlarged, a feature that is shared with erythrocytic stages of *P. falciparum *and *P. malariae*. Distorted infected erythrocytes, which are a common feature in *P. knowlesi *infections of rhesus macaques [13,14], were rarely observed in human *P. knowlesi *infections in the present study. No stippling was apparent that resembled Schüffner's dots, which are observed in *P. vivax *infected erythrocytes. However, faint stippling that appeared as light irregular dots were evident in only some of the infected erythrocytes with mature trophozoite and schizont stages. This type of stippling in erythrocytes infected with *P. knowlesi *is referred to as 'Sinton and Mulligan's stippling' and was noted previously in infections in rhesus monkeys [1,14,16] and humans [18]. According to Sinton and Mulligan [14], who first described this, the stippling was only consistently observed in some infected erythrocytes of macaques, when the panoptic method of Green [19] was used for staining. Similarly in *P. malariae *infections, the infected erythrocytes are not enlarged or distorted and may display some form of irregular fine stippling in blood films with intense staining [1].

Morphologically, and depending on the predominant circulating parasite stage, it was not possible to identify robust morphological differences to distinguish between *P. knowlesi *and *P. malariae *or *P. falciparum *using routine microscopy methods for malaria diagnosis. This lack of distinguishing features accounts for *P. knowlesi *infections being overlooked, despite major differences in the pathogenesis of each of these different lineage parasites [4,20]. Laboratory misidentification of *P. knowlesi *as *P. malariae *by microscopy is largely unavoidable, although it is difficult to rationalize how a parasitaemia greater than 100,000 per μl blood, together with signs of severe malaria, could clinically be mistaken for a *P. malariae *infection, except that until recently, such infections would have been relatively rare and on a background of *P. falciparum *transmission. Until the application of DNA based technology, the definitive identification test for *P. knowlesi *parasites was to inject infected blood into rhesus monkey and observe for a fatal outcome [15]. Clearly this was, and is beyond the scope of all but a few international centres with this type of facility. Readily available molecular tools, most notably the *P. knowlesi*-specific PCR assay [3], provide a relatively available means to properly identify *P. knowlesi *[3,6-9]. Molecular methods are expensive, not available in most rural diagnostic laboratories and as such, are not currently used for routine malaria diagnosis [21]. Areas of Southeast Asia, where there is a suspicion that *P. knowlesi *infects humans, would benefit from a PCR screening exercise to estimate the relative proportion of *P. knowlesi *to *P. malariae *infections. This information would be valuable to prime local healthcare professionals to recognize severe knowlesi malaria in ill patients with a laboratory report of *P. malariae*.

Despite morphological similarities with *P. malariae*, there are other clinical and parasitological parameters such as presentation with one or more of the WHO clinical criteria for severe malaria and a parasitaemia greater than 5,000/μl blood, that, together with a history of time spent in the forest and forest fringe areas of Southeast Asia, should strongly implicate a *P. knowlesi *rather than a *P. malariae *infection [4,20]. Given the consequence of misdiagnosis and delayed treatment for this parasite with a relatively short erythrocytic cycle, every effort should be made to accurately diagnose knowlesi malaria in populations at risk.

## Conclusion

The morphology of *P. knowlesi *parasites in human infections closely resembled those of *P. falciparum *in the early trophozoite stage and *P. malariae *in the later stages of the erythrocytic cycle. Although there were some minor morphological differences between the blood stages of *P. knowlesi *and *P. malaria*e, it would be difficult to positively identify knowlesi malaria based on morphology alone. However, even in the absence of PCR facilities, severe symptoms, a parasitaemia >5,000/μl blood, with *P. malariae *parasite morphology and a recent history of time spent in the forest fringe areas of Southeast Asia should be enough to make a diagnosis of knowlesi malaria. In view of what is now known of the distribution and severe manifestation of knowlesi malaria, continued misdiagnosis of this important pathogen is no longer acceptable.

## Competing interests

The authors declare that they have no competing interests.

## Authors' contributions

KSL, BS and JCS wrote the paper, BS and JCS conceived the study, KSL stained and examined the slides by microscopy.
